# Digital focus groups in healthcare interventions: A reflexive methodological analysis of how socio-technical and relational factors co-produce qualitative data

**DOI:** 10.1186/s12874-026-02945-7

**Published:** 2026-07-11

**Authors:** Jan Gehrmann, Johannes Stephan, Ananda Stullich, Julia Roick, Laura Hoffmann, Matthias Richter

**Affiliations:** 1 TUM School of Medicine and Health, Department Health and Sport Sciences, Social Determinants of Health, Technical University of Munich, Munich, Germany; 2 TUM School of Medicine and Health, Department Clinical Medicine, Institute of General Practice and Health Services Research, Technical University of Munich, Munich, Germany

**Keywords:** Focus Group, Qualitative Research, Data Collection, Research Design, Digital Divide, Group Processes, Qualitative Inquiry

## Abstract

**Background:**

Digital focus groups are increasingly used in health research, yet their methodological implications within complex interventions remain insufficiently examined. When conducted with pre-existing cohorts, digital focus groups may function not only as evaluative tools but also as relational and socio-technical encounters that shape the generation of qualitative data. The goal of the present study was to examine how digital focus groups operate within this context.

**Methods:**

This study presents a methodological re-analysis of focus group material originally collected for the evaluation of a hybrid psychosocial prevention intervention, examining how digital focus groups operate within this context following the perspective of Situational Analysis. Digital focus groups were conducted with participants who had completed the hybrid intervention together in fixed cohort groups, meaning participants were already acquainted with one another prior to the focus group sessions. For the analysis, the transcripts were examined using descriptive, inductive coding. Verbatim quotations were used as methodological markers to illustrate how relational, emotional and technological factors shaped the data.

**Results:**

Pre-existing cohort cohesion strongly influenced how participants engaged in the digital setting, fostering emotional safety, peer-supported learning, motivational reinforcement and opportunities for self-regulation. Digital platforms structured visibility, turn-taking and synchronization, while differences in devices, connectivity and digital literacy affected participation and the stability of group interaction. The researcher role expanded to include facilitation, technical troubleshooting and emotional anchoring, making the research team part of the socio-technical infrastructure. At times, the focus groups elicited supportive or motivational effects that echoed elements of the intervention itself, blurring the line between evaluation and unintended interventional effects.

**Conclusions:**

Focus groups in intervention research operate as relational encounters rather than neutral data-collection tools. This applies particularly to Digital Focus Groups. Recognizing how pre-existing relationships, technological environments and researcher involvement shape interaction is essential for methodological transparency and rigorous interpretation. The findings highlight the importance of reflexivity, attention to digital differences, and awareness of unintended interventional dynamics when designing and interpreting digitally mediated qualitative methods in healthcare contexts. Furthermore, the findings indicate that the choice between digital and in-person FGs should not be guided by pragmatic considerations alone, but by the relational, technological and institutional conditions of the research situation.

**Trial registration:**

DRKS-ID: DRKS00033080 (registration date: December 7, 2023).

## Background

Focus Groups (FGs) are among the most established and widely valued methods in qualitative research [1–4]. With their capacity to generate interactionally produced insights, they offer researchers a unique view into collective meaning-making, shared experiences, and relational processes that extend beyond what individual interviews can elicit [4, 5]. Their long-standing use across disciplines attests to their methodological robustness and their distinctive ability to shed light on how participants negotiate, co-construct, and sometimes contest interpretations together.

As qualitative research increasingly moves into digital environments, however, it becomes essential to rethink how this established method transforms when relocated to digital spaces [6–10]. Digital Focus Groups (DFGs) are often treated as pragmatic adaptations of in-person encounters, yet their epistemic, relational, and material dynamics are widely discussed [11–15]. This is particularly salient when technologies not only mediate the research encounter but are also integral to the intervention under study. In such cases, DFGs function as socio-technical events in which platforms, infrastructures, participants and researchers jointly shape what can be articulated and how knowledge is produced [11, 13, 16–18]. It is important to note that some of these relational dynamics such as pre-existing group cohesion and unintended supportive effects may occur in longitudinal or cohort-based focus groups regardless of format. What the digital setting distinctively contributes is the mediation of these dynamics through platform affordances, device differences, and technologically structured interaction, all of which may shape and intensify their expression. These dynamics further suggest that, within intervention-linked qualitative research, the distinction between evaluation and unintended supportive or unintended interventional dynamics (such as self-reflection, re-experiencing, shame, distress or uncertainty) may become blurred [19–22].

Therefore, it is important to analyze these dynamics in order to better understand and interpret participants’ narratives, emotional responses, and the ways in which the data collection situations itself may shape the data in digitally mediated health research.

The reflexive methodological approach taken in this study is grounded in a multi-site research project accompanying the development of a hybrid, app-supported psychosocial prevention program completed by participants in stable group-based cohorts [23]. Although the DFGs conducted within this project were designed as an evaluative component [23, 24], it became evident as the sessions unfolded that their function extended beyond data collection: they also initiated reflections and supported participants’ ongoing behavioral change. This methodological re-analysis is informed by perspectives from Situational Analysis (SiA), which conceptualize qualitative research encounters as socio-technical and relational situations rather than neutral sites of data elicitation [25].

By situating this reflexive methodological analysis within concrete empirical material, the study contributes to current methodological discussions on data quality, participation equity and the design of qualitative components in digital health research. Accordingly, it addresses the following research question: *How do DFGs embedded in hybrid clinical interventions operate as socio-technical and relational research situations*,* and how do these conditions shape participation*,* interaction and the co-production of qualitative data?*

## Methods

### Study background and setting

The DFGs were embedded within the larger research project “Development, piloting and evaluation of an app-supported psychosocial prevention intervention” (German acronym: PE³PP), funded by the Federal Ministry of Labour and Social Affairs. The Technical University of Munich (TUM) is responsible for the scientific evaluation. The study protocol gives an overview of the overall Mixed-Methods study design and the project itself [23].

**Fig. 1:**
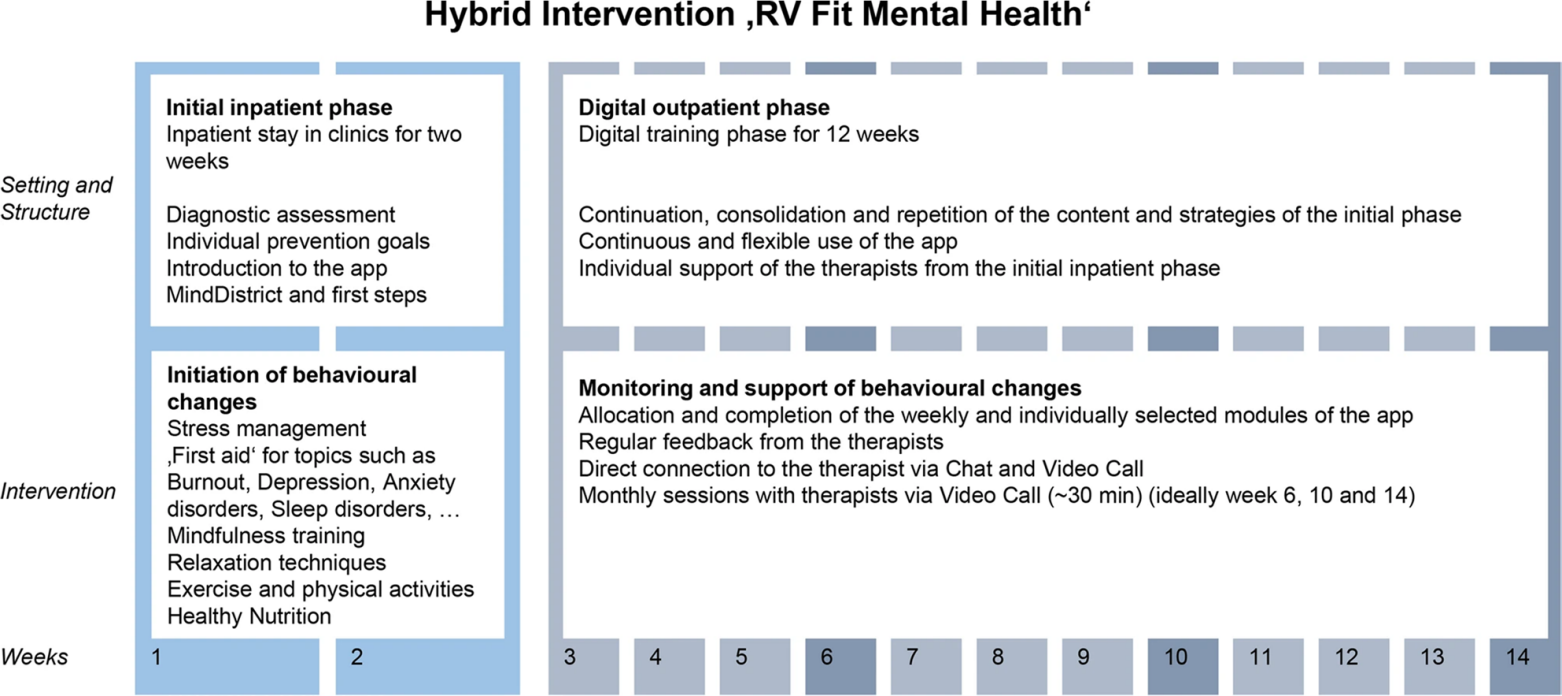
Visual overview of the intervention structure, including the two-week inpatient phase and the 12-week app-supported outpatient phase. Reproduced from Gehrmann et al. 2026 [26]

The intervention targets psychological, psychosomatic, and psychosocial aspects of work-related mental health and participation restrictions. It consists of a 14-week hybrid structure, combining a two-week initial inpatient phase with a 12-week digital outpatient phase, during which participants work through app-based modules and receive continuous therapeutic support (Fig. 1). Trial participation is organized in cohort groups of up to ten individuals and several modules in the inpatient phase are delivered in group settings, so that the social and therapeutic relationships formed during the inpatient phase carry over into the subsequent digital phase. As a result, participants already knew each other on a more personal and in-depth level when taking part in the DFGs, which shaped the interactional dynamics and depth of the discussions. The study protocol provides a full overview of the mixed-methods evaluation design [23]. The pilot study supported the feasibility of the DFG format within this intervention context and informed the refinement of the discussion guide used in the present study [24]. The substantive findings of the evaluation, including participants’ experiences of the intervention and its effects, are reported in separate publications. The present paper focuses exclusively on the methodological conditions under which the DFGs operated as research situations.

### Study design

This study constitutes a qualitative methodological approach based on a re-analysis of DFGs transcripts generated within the PE³PP evaluation study. While the original evaluation aimed to explore participants’ experiences with a hybrid psychosocial prevention intervention [23, 24], the present study examines the DFGs themselves as socio-technical and relational research settings. Reflexive methodological re-analyses and qualitative secondary analyses constitute an established and peer-reviewed form of inquiry within qualitative health research, particularly when the analytical focus differs substantially from that of the original study [27–30]. Conceptually, our reflexive methodological analysis draws on two complementary but distinct frameworks. The primary conceptual anchor is the socio-technical perspective as developed in digital qualitative and digital health research [6, 7, 16], which foregrounds the co-production of knowledge through the interplay of human actors and technological systems — including platforms, devices, and connectivity infrastructures. This is supplemented by socio-material perspectives [31], which emphasize the mutual constitution of social practices and material artifacts in institutional settings. While these perspectives overlap, they are not interchangeable: the socio-technical frame organizes our analytical focus on how platform affordances and digital infrastructures shape research interaction, while the socio-material perspective informs our attention to the constitutive role of non-human actors such as devices and interfaces in the research situation. Both perspectives are grounded within the broader SiA framework [25, 32], which conceptualizes research encounters as heterogeneous situations composed of human and non-human actors, discourses, and material infrastructures. Although we did not apply formal situational mapping techniques, our analytical logic follows the core premise of SiA, namely to render visible the heterogeneous elements and relations that constitute the research situation and co-produce qualitative data [25]. Previous empirical studies have shown that SiA is well suited for examining digitally mediated and institutionally embedded research settings, as it conceptualizes data generation as the outcome of heterogeneous socio-technical constellations rather than as the product of isolated individual accounts [33, 34]. Such an approach helps to make visible how interactional dynamics, technological infrastructures and organizational arrangements jointly shape participation and co-production of qualitative data.

For the purposes of this analysis, all DFG transcripts were used in their entirety. The transcripts were not re-sampled or filtered prior to analysis; rather, the full dataset was re-examined with an analytical focus that differed from the original evaluation study. While the original analysis was directed at the substantive content of participants’ experiences with the intervention, the present analysis directed attention exclusively to the interactional, relational, and socio-technical conditions under which those statements were produced. This shift in analytical focus, from content to situation, is what constitutes the methodological re-analysis and justifies the use of the same dataset for a distinct research question. The dataset of nine DFGs, comprising participants across two clinical sites and spanning approximately 21 months, provided an empirically diverse basis for examining recurrent situational patterns within digitally mediated intervention contexts [35, 36]. So, no additional data were collected for this methodological analysis. This study is reported following the consolidated criteria for reporting qualitative research (COREQ) [37, 38].

### Data collection

Primary data collection within the evaluation study was conducted as follows: Participants were recruited by the clinical staff, and were provided with the study information, consent forms and dial-in details for the DFG. A detailed step-by-step instruction sheet for using the platform was also distributed and participants were offered the option of an individual test run prior to the session to familiarize themselves with the technical setup [39].

DFGs were conducted between January 2024 and October 2025 comprising a total of 32 participants across all groups, scheduled at varying intervals to capture different stages of the project. DFGs lasted on average 85 min (Min.: 64; Max.: 113). To ensure a stable interactional environment, all sessions were co-facilitated by two researchers: one guided and moderated the discussion, while the second acted as technical support and monitored audio/video issues. The dual role assumed by researchers in these sessions, simultaneously moderating the discussion, managing technical difficulties, and providing emotional orientation, warrants explicit reflexive consideration. This configuration meant that researchers were active participants in the socio-technical infrastructure of the DFGs. Their interventions, whether directing turn-taking, verbalizing nonverbal reactions for the audio record, or guiding participants through the platform interface, shaped the interactional conditions under which data were produced. It is likely that this expanded researcher presence contributed to the sense of safety and structure that participants reported, but may equally have influenced which topics were raised, how openly participants spoke, and how group dynamics unfolded. These effects cannot be fully disentangled from the data, but reflexive awareness of the researchers’ constitutive role in the digital research situation is itself part of the methodological transparency this study seeks to model.

Sessions were audio-recorded through an additional laptop. Zoom was used for digital sessions due to an existing university license. Participants were not familiar with the researchers prior to the DFGs. Participants usually joined the video-based sessions from their homes, typically alone in the room while in some cases supporting relatives or partners were present for technical support. All participants used their camera; the chat function was only used to distribute information about technical support (like phone numbers). Recordings were transcribed verbatim and pseudonymized by an external professional service following standardized transcription rules. The transcripts were produced as verbatim content transcripts that also included interactional features such as interruptions, overlaps and references to technical disturbances. The DFGs were conducted in German. Participants communicated in German throughout all sessions, though a minority of participants did not speak German as their first language. The quotations presented in this paper are translations into English prepared by the research team, with attention to preserving the interactional character, colloquial register, and emotional tone of the original utterances. Non-verbal reactions (e.g. nodding, facial expressions) were verbally explicated by the moderators during the sessions if relevant and were therefore captured in the audio recordings and subsequent transcripts. In addition, unstructured field notes were taken by the researchers during the FGs and a peer debriefing took place to discuss shared reflections on the DFGs. Transcripts were not returned to participants for review.

The DFGs guide was developed through a structured, multi-step process aligned with established qualitative research standards [4, 5, 40, 41]. Based on the study’s aims and research questions, thematic areas were identified and translated into open-ended, neutral prompts designed to elicit detailed responses and encourage active discussion. Each session concluded with a short specifically moderated Wrap up, a brief closing round in which each participant was invited to offer final comments or reflections on the DFGs. The guide was iteratively refined within the research team to improve flow, remove redundancies, and ensure clarity and comprehensibility. No stimulus material was used, as preliminary DFGs in the pilot study (October 2023-March 2024) had shown limited value of stimulus material [24]. Table 1 shows the self-reported characteristics of the sample across the DFGs.

**Table 1:** Individual participant characteristics across the DFGs

Category	Value (*n* = 32)
Age in years, mean (Min.; Max.)	51.7 (37; 64)
Gender (%)	
Female	65.6
Male	34.4
Migration background first or second generation (%)	12.5
Education ISCED groups (%)	
Low education (No or basic secondary education)	3.1
Medium education (intermediate or upper secondary education)	28.1
Vocational education (Completed vocational training)	43.8
Tertiary education (Under- or postgraduate, or doctoral degree)	25.0
Place of Residence (%)	
Rural area	25.0
Small town	21.9
Medium-sized or large city	53.1
Chronic Disease (%)	81.3
Subjective Work related Health risk (%)	
Very strong	6.2
Strong	12.5
Moderate	62.5
Not at all	18.8

### Data analysis

The present methodological re-analysis is independent of the analyses conducted in the evaluation. For the purposes of this methodological study following the principles of SiA, the DFGs transcripts were re-analyzed not to identify content-related themes, but to analyze how DFGs function as socio-technical, relational and interactional research settings. This approach draws on established perspectives that conceptualize FGs as sites of collective meaning-making and interactional co-construction [5, 41, 42], and qualitative data collection as actively shaped encounters between researchers and participants [43]. It also aligns with socio-material and digital qualitative research approaches that emphasize the co-presence of technologies, settings and human actors in shaping digital environments [31, 44–46]. For this study, a first-cycle inductive coding was used to identify segments in which relational, technological, emotional and procedural aspects of the research situation became visible. Codes did not represent thematic content of the intervention but features of the interactional and socio-technical conditions under which statements were produced (e.g., references to platform affordances, turn-taking, peer support, researcher intervention, emotional anchoring, or infrastructural disruptions). Coding followed established procedures for descriptive, text-close coding [47] and semantic inductive coding used in thematic analysis [48]. In a second step, these codes were analytically related to each other to reconstruct the broader situation of the DFGs, following the logic of SiA [25, 32]. Rather than building a category system or theory, the aim was to make visible how heterogeneous elements of the situation formed a constellation that structured participation, interaction and meaning-making. To enhance analytic rigor, reliability and intersubjective accountability, the coding and interpretation process was accompanied by regular reflexive discussions within the research team. Preliminary codes and analytic memos were reviewed and critically discussed by multiple researchers, and divergences in interpretation were resolved through consensual reflection rather than formal intercoder reliability testing. This procedure aimed to ensure transparency, coherence and reflexive awareness of how analytic decisions were made and how the situational and socio-technical dimensions of the data were interpreted [49–52].

### Reporting and quotation strategy

To illustrate the methodological phenomena discussed in this paper, selected excerpts from the DFGs transcripts are presented. The inclusion of verbatim quotations serves not merely as illustration but as a methodological tool for transparency, reflexivity and auditability in qualitative research [53]. Lingard [54] emphasizes that quotations should be selected purposefully to support coherence and credibility rather than function as isolated fragments. Eldh et al. [53] similarly note that the rationale for quoting is often under-reported and argue that excerpts should illuminate the analytic process rather than merely validate findings. In this study, quotations help make relational, technological and emotional dynamics visible. By presenting excerpts with pseudonyms, identifiers and transcript positions, the quotation strategy responds to calls for transparency, contextualization and traceability in qualitative reporting [37, 38, 53, 55, 56]. Used in this way, quotations expose not only content but also context - how platform affordances, moderation practices and cohort dynamics shaped the discussion. This reflects broader arguments that quoting is never neutral but a situated and reflexive methodological choice [55, 57, 58]. The excerpts presented in this study were selected because they represent dynamics that were observable across multiple DFG sessions and multiple participants - not as isolated instances but as recurrent features of the research situation. Where a dynamic was visible in only one or two sessions, this is noted explicitly. The selection process was documented in analytic memos and discussed within the research team as part of the reflexive coding procedure described above.

All quotations are shown with pseudonyms of participants (P) or interviewers (I), a randomly assigned Focus Group identifier (FG-ID), and the respective transcript position (Pos).

## Results

Across the DFGs, five interrelated dimensions were observable: (1) the influence of pre-existing cohort cohesion, (2) interactional processes that coincided with unintended interventional dynamics, (3) platform-specific conditions shaping turn-taking and visibility, (4) digital and infrastructural differences, and (5) shifts in researcher roles within the online environment. All five dimensions were observable across multiple sessions and both clinical sites. Their relative salience and intensity varied across cohorts, reflecting differences in group composition, timing of the DFG relative to the intervention, and participants’ technical infrastructure. This variation is itself a methodologically relevant finding, as it illustrates how the specific configuration of each research situation shapes the dynamics of data production.

### Dimension 1: Peer dynamics and pre-existing group cohesion

Pre-existing cohort cohesion was observable to varying degrees as an interactional feature across all DFG sessions, although its intensity differed depending on the timing of the session relative to the end of the inpatient phase and the composition of individual cohorts. Across the DFGs, participants drew on their shared history as intervention cohorts. Rather than forming a new group in the research setting, they entered the digital sessions with prior relational familiarity originating from the hybrid psychosocial program. This manifested in repeated references to earlier shared experiences and in interactional patterns that built on pre-existing connections. This dynamic was evident in how participants framed the DFGs as an opportunity not only to provide evaluative feedback but also to reconnect on a relational level. For some, the session generated a sense of reunion, of briefly returning to a familiar cohesion created during the intervention:

*„(…) it [the DFG] was like coming home*,* wasn’t it? As if we hadn’t been away at all. And I also thought it was good that we/everyone calmed down a bit. Everyone had time to let everything sink in. And I think everyone took something away from it and contributed to it.“ (FG-ID4*,* Pos. 713)*.

Group cohesion was also visible in routine interactions. Participants spontaneously exchanged logistical information, such as sharing a message from an absent group member within the DFGs, which demonstrated an ongoing sense of interpersonal orientation within the cohort. This moment reveals how the group maintained a shared sense of communal responsibility:

*„P1: Yes*,* (first name of P3)*,* please read out what (first name of the absent participant) wrote.*

*P3: (First name of the absent participant) wrote: “Please excuse me*,* I was at the hospital until just now and didn’t have my cell phone with me.” (FG-ID4*,* Pos. 508–509)*.

Several participants reflected on the relevance of past group-based intervention components, noting that learning processes during the program had been closely tied to peer exchange:

*„Well*,* I found everything very exciting*,* but what really stuck in my mind was the group therapy*,* that it was actually the best thing*,* or that I gained a lot of insights through the group therapy*,* through being together*,* through observing the other patients and their insights*,* and that this actually strengthened me the most and helped me the most.“ (FG-ID3*,* Pos. 284)*.

In some cases, the DFGs appeared to prompt considerations of re-establishing or continuing contact beyond the research setting:

*„Yes*,* it was nice to hear from everyone again. You really feel guilty for neglecting the [Messenger] group. That’s just how it is. Maybe we should organize a class reunion at the end of the summer so we can all see each other again. Maybe we can meet up somewhere again?“ (FG-ID6*,* Pos. 302)*.

However, cohort cohesion was not a uniform or uncontested condition across all sessions. Dissonant, asymmetrical or uncomfortable dynamics also occurred, and are analytically revealing because they illustrate that pre-existing relational familiarity is a contingent achievement rather than a neutral backdrop. In one group, a participant had been unable or unwilling to engage with the shared relational space of the cohort, and withdrew from further contact entirely following the sessions:

*“We had one participant who did not want to open up*,* could not. I don’t know. I don’t want to judge. There is no contact with her at all anymore*,* because she doesn’t want that either. […] We also have a male participant who is in shift work in care*,* and we try to maintain contact with him.” (FG-ID3*,* Pos. 128)*.

This moment illustrates that cohort cohesion is produced unevenly: while some participants experienced the DFG as a space of reconnection and mutual recognition, others remained at the margins or disengaged entirely. Such differential participation directly shapes the interactional texture of the session and the range of perspectives that enter the data. Finally, subtler forms of relational positioning were also present, such as moments of social comparison expressed through humor:

*“P1: Well*,* I’m probably the swot here*,* right?*

*P2: Yes, always. Always, [name of P1]. You’re always the swot.” (FG-ID3, Pos. 58–59)*

While this exchange was handled with humor and quickly normalized by the group, it points to the presence of evaluative undercurrents within the cohort dynamic — a form of relational positioning that shaped how participants framed and presented their own experiences during the discussion.

### Dimension 2: Unintended interventional dynamics

While dynamics that extended beyond the evaluative purpose of the sessions were observable in six of the DFGs, the sense of reconnection and coming together among cohort members was a consistent feature across all sessions. Across several DFGs, participants described the discussions as relating not only to the evaluation of the intervention but also to aspects that resembled interactional dynamics from the program itself. Individuals referred to the exchange as helpful for situating their own experiences in relation to those of others, and some emphasized that hearing about similar challenges from fellow cohort members remained meaningful in the post-intervention phase:

*„I also love seeing you all again and hearing how you’re coping with things. It makes you feel less alone*,* doesn’t it? You’re all facing similar challenges*,* and that makes me feel good.“ (FG-ID6*,* Pos. 305)*.

Such statements indicate that the DFGs provided a setting in which participants related their ongoing experiences to those of their peers and revisited reflections they associated with earlier stages of the intervention. Across the dataset, participants articulated a range of dynamics that extended beyond the immediate evaluative purpose of the sessions, including opportunities for shared orientation, mutual reassurance and revisiting personal strategies (see Table 2). These observations suggest that the DFGs created reflective conditions that participants associated with processes supporting behavioral change during the program. It is important to note that these dynamics are not claimed to be inherent to DFGs as a methodological format. Rather, they appear to arise from a specific configuration of conditions: DFGs conducted with pre-existing cohorts who share a therapeutic history, at a point in time when participants remain embedded in an ongoing intervention process. Whether comparable effects would emerge in DFGs conducted with newly formed groups, in post-intervention follow-up contexts, or outside of psychosocial intervention settings remains an open question that the present study is not positioned to answer.

**Table 2:** Dimensions of unintended interventional dynamics

Dimension	Illustrative quote
DFGs as peer-supported learning processes and capability building The DFGs enabled participants to revisit concerns, compare experiences, and articulate what had worked or remained difficult. The group provided orientation and the sense that personal insights could be developed collectively.	*„Well*,* I thought it was really nice that we the three of us together again and were able to discuss our concerns once more in order to get more feedback: What went well during the entire phase? What didn’t go so well during the phase? Then you can say or hope that it will be passed on and you can make the best of the whole situation*,* right?“ (FG-ID4*,* Pos. 715)*
DFGs as motivational reinforcement and behavioral prompting Some participants described the DFGs as timely situated as the session reinvoked earlier commitments and created an opening to reconnect with personal goals. The goals of the interventions were also brought back into mind (like scheduling coaching sessions).	*„that we are doing this [the DFGs] now*,* in a period where we basically still have a few weeks left*,* so not just at the end*,* but as a reminder*,* so to speak.“ (FG-ID6*,* Pos. 301)*
DFGs as reflective self-regulation processes Some used the DFGs to revisit internal processes that rarely became shared topics in everyday life. The DFGs offered a setting in which personal issues could be articulated and reconsidered in relation to others.	*„Yes*,* it was interesting for me to talk about things again that I usually only discuss with myself. Things that are there and present but aren’t normally the subject of conversation. And that’s something that happens rather rarely and*,* in my opinion*,* is easier in a group like this (…). And it was actually quite good that I was able to think about myself a little bit again and reflect on it.“ (FG-ID8*,* Pos. 136)*
DFGs as affective support and regulation Some experienced the DFGs as a stabilizing space that eased uncertainty and reintroduced a sense of connection. The session provided reassurance, reduced feelings of being alone with challenges, and created an atmosphere in which emotional responses could be acknowledged collectively.	*„Well*,* I was actually a little worried today that everything would come back up again*,* but I was happy to see everyone again and really enjoyed it.“ (FG-ID5*,* Pos. 313)*

The DFG format also surfaced a structural difference in intervention delivery that one participant had been unaware of — specifically, that the app-based modules were not delivered uniformly to all participants, but were individually tailored and continuously adjusted by the therapist in response to each participant’s progress and needs. This personalization of content, a feature of the intervention that other participants in the same session had not experienced or been informed of, became visible during the discussion:

*“P2: (…) we actually talk about the contents of the app*,* whether there is anything going on in my everyday life that is on my mind right now*,* and where he might look for a new module that fits. These conversations are really also about looking at: what modules would be worth offering me? Because otherwise it stays quite general.*

*P3: Well*,* I have to say*,* [name of B2]*,* this is the first time I’m hearing this. I really hadn’t assumed that we would talk through the modules individually. [Therapist] somehow didn’t get that across to me that way.” (FG-ID3*,* Pos. 218–219)*.

This asymmetry was further underscored in a different session, where a participant realized mid-discussion that she had not received any therapeutic contact at all during the app-based phase:

*“In my case*,* I am wondering right now*,* I have not had a single conversation yet*,* not even an offer of a conversation. So that is a little strange.” (FG-ID6*,* Pos. 145)*.

These exchanges illustrate how the DFG created a shared interactional space in which differences in intervention delivery became visible not through researcher questioning, but through participant-to-participant exchange. Such moments would likely have remained invisible in individual interviews and point to the DFG’s capacity to surface intervention-level variation as an unintended but methodologically revealing outcome of the group format. Notably, the disclosure in the first exchange prompted an immediate shift in one participant’s intended behavior — P3 announced she would now seek contact with her therapist — illustrating how DFGs can blur the boundary between evaluation and unintended intervention not only relationally but also at the level of participants’ engagement with the intervention itself.

### Dimension 3: Technological constraints

Across the DFGs, participants’ engagement with the discussions was shaped by the digital environment in which the sessions took place. These platform-mediated dynamics were observable across all DFG sessions, though their salience varied depending on the technical setup of individual participants and the stability of connections on a given day. The platform structured how participants appeared to one another, how they managed turn-taking, and how conversational flow developed. Several participants contrasted the video-based exchange with written communication, noting that seeing each other on screen created a different form of interaction, generated a sense of presence that text-based channels could not reproduce:

*„That was great. Just a nice refresher. We saw each other again and it felt different than when we just write or talk to others about it.“ (FG-ID7*,* Pos. 449)*.

Participants also retrospectively reframed technical difficulties as shared challenges that the group had collectively overcome, transforming moments of potential disruption into markers of interactional resilience. Interactional rhythms were closely linked to the stability of the technical setup. Instances of lag, absent video, or initial uncertainty became part of the unfolding conversation, influencing how the group synchronized and proceeded:

*„It was a great success*,* even though there was a bit of excitement beforehand about whether everything would work out (…) and we didn’t have any screens in between and so on*,* but we managed everything*,* didn’t we?“ (FG-ID4*,* Pos. 726)*.

### Dimension 4: Digital divide and infrastructural differences

Access to the digital space varied considerably across participants. Differential access was apparent in at least five sessions, ranging from brief technical disruptions to cases in which individual participants were unable to maintain stable video connections throughout the discussion. Differences in connectivity, device type and digital literacy shaped who was able to join, how continuously individuals could participate and how easily they could navigate the session. These factors influenced the composition of the groups and affected the stability of interaction throughout the discussions. Some participants entered the session with uncertainty about their technical setup, unsure whether they would be able to follow or contribute. The digital setting created a layer of tension that sat alongside the emotional dimension of reconnecting with the group and to the past “flow” of the inpatient phase:

*„I am pleasantly surprised. I was a little nervous about both the technical aspects and the content*,* and wondered whether this would be another one of those events where everyone just sits silently in front of six screens*,* but I am*,* yes*,* pleasantly surprised that we managed to get back into the flow so well together. And yes*,* as I said before*,* I have found the program very*,* very valuable for me so far*,* both in terms of content and on a personal level*,* from therapists to fellow patients. As I said*,* it has definitely added value for me.“ (FG-ID5*,* Pos. 308)*.

In other groups, participants commented on the absence of peers who had encountered technical barriers, indicating an awareness that differing levels of access and digital competence shaped who could be present. The limits of digital literacy or unstable connections were sensed not only technically but relationally:

*„Yes*,* I thought it was a thoroughly successful event and would have been delighted if the others had been there too*,* but as (first name of another participant) said*,* we had almost feared that it wouldn’t go so well.“ (FG-ID6*,* Pos. 228)*.

Technical constraints also shaped how participants could visually orient themselves within the group. Device-related differences affected what could be seen on screen and how readily participants could follow the discussion, often leading to troubleshooting exchanges within the session:

*„I1: We still have a recording running here*,* so you may see that another PC is logged in. It’s called the recording PC. My colleague [name of I2] also has it at home. Just so you know that we are recording this. Exactly. Now you can see a second screen with (first name of I2)*,* if you can see us all*,* perhaps.*

*P4: I can’t see anything.*

*P3: I can only see one.*

*P2: Yes*,* me too. Now I can see (first name of P3).*

*P1: I don’t know about cell phones*,* but go to “Layout” and then “Gallery.” Then you’ll always be able to see everything.*

*P3: Exactly, I only have a cell phone.“ (FG-ID6, Pos. 2–7)*

At times, these barriers led to interruptions in participation or even prevented individuals from remaining in the session. These moments made the influence of infrastructural differences on participation visible:

*„It was very well moderated*,* even though half of the participants lost interest and dropped out. No*,* I think there were technical challenges. But yes*,* it was great.” (FG-ID8*,* Pos. 140)*.

It is worth noting that the phrase ‘lost interest and dropped out’ in this excerpt represents the participant’s initial interpretation, immediately self-corrected to ‘technical challenges’. This moment of self-correction is itself methodologically instructive: it illustrates how technical exclusion can be misread as disengagement even by those present in the session, pointing to the relational and interpretive consequences of infrastructural barriers beyond their immediate practical effects. Participants joined the sessions from their home environments using a range of devices (including smartphones, tablets, and laptops or desktop PCs) and these differences directly shaped visibility and navigability within the platform. Prior to each session, all participants had received a detailed step-by-step instruction sheet and were offered an individual test run with the platform. That technical difficulties nonetheless occurred across multiple sessions underscores that infrastructural barriers in digital qualitative research cannot be fully mitigated by preparatory measures alone, and that device diversity and connectivity variability constitute persistent sources of interactional asymmetry.

### Dimension 5: Shifting researcher roles

In the DFGs, researchers assumed responsibilities that extended beyond conventional moderation. This expanded role was observable across all DFG sessions and is documented in both the transcripts and the researchers’ field notes. A basic form of role differentiation was already built into the study design itself, with one researcher leading the moderation and discussion while the second acted as technical support — a division that made the expanded and multifaceted nature of researcher involvement in digital settings structurally visible from the outset. Notably, this role differentiation was not only a background condition but was at times explicitly noticed and commented on by participants themselves. The online environment required them to combine facilitation with technical guidance and explicit management of interactional cues. The flow of the conversation was influenced by how researchers responded to technical interruptions, navigated limited nonverbal communication, and oriented participants within the digital setting.

Participants commented on the moderation, noting that the balance between structured questioning and a relaxed conversational style supported their engagement:

*„P2: (…) it was great*,* just because we were more relaxed*,* constructive*,* maybe also because we were on first-name terms*,* I don’t know if that helped too.*

*P1: Yes.*

*P2: I thought it was very good. And I noticed that there’s definitely something intention behind it. You have to ask certain questions*,* but it was quite relaxed. And you were given time and space. That’s why it was a very pleasant conversation.“ (FG-ID1*,* Pos. 152–154)*.

Researcher visibility also formed part of the interaction, as gestures and attentiveness were noticed by participants and factored into how they interpreted the discussion:

*„You*,* (first name of I1)*,* I have definitely noticed that you listen very well and nod*,* and I see (first name of I2) nodding too*,* sometimes*,* but of course I don’t hear much from (first name of I2).“ (FG-ID8*,* Pos. 140)*.

This observation illustrates how researcher visibility in the digital setting operated differently from in-person FGs: participants actively monitored and commented on researchers’ nonverbal cues on screen, making the researcher’s embodied presence a visible element of the interactional dynamic rather than a background condition. The digital setting appeared to render the researcher a more conspicuous socio-technical actor whose visibility was both mediated and scrutinized through the platform. Because nonverbal communication was limited on screen, researchers intervened more explicitly to distribute speaking opportunities and include quieter participants:

*“I1: (First name of P1)*,* (First name of P3)*,* brief impression of you?” (FG-ID6*,* Pos. 48)*.

Technical differences between devices also shaped participation, and researchers responded by addressing participants individually, guiding them through the interface and orienting them within the session:

*„I think it should be easier for (first name of P2)*,* (first name of P1)*,* and (first name of P5) on the PC. (First name of P3) and (first name of P4)*,* something should have popped up on your screen by now.“ (FG-ID6*,* Pos. 312)*.

In situations where nonverbal reactions were not captured on the audio track, researchers verbalized what they observed on screen to ensure clarity and shared understanding so that participants were aware of mutual reactions:

*„I1: You’re all nodding. (Agreement) I always have to comment on things like that because we only have the audio track.*

*P2: Oh*,* right*,* exactly. Yes*,* no*,* exactly.*

*I1: That means when everyone nods*,* exactly*,* I always have to say so briefly.*

*P1: Yes*,* really everyone [nods]. Yes.“ (*FG-ID*3*,* Pos. 83–86)*.

## Discussion

FGs have long been used to access shared sense-making, negotiated meanings, and interactional knowledge [2, 42, 59]. Their epistemic strength lies in enabling participants to respond to one another, build on emerging ideas, and articulate experiences that gain shape only in relation to others [5]. Classic qualitative work has shown that FGs are not neutral settings but relational and interactional encounters in which meaning is co-produced through conversation and situated forms of participation [60, 61]. From a situational-analytic perspective, the DFGs can be understood as arenas in which multiple social worlds intersect, including the intervention context, the digital platform, and the research setting [25, 32, 62]. In the DFGs examined in this study, these dynamics were shaped not only by interactional processes but also by the specific socio-technical conditions and by pre-existing cohort dynamics, platform-specific affordances, and the emotional and practical conditions of digital participation (as it is mentioned especially in digital ethnography approaches) [44, 63–65].

Participants entered the sessions with shared histories and relational familiarity formed during the hybrid psychosocial intervention. This foundation influenced how interaction unfolded, how openly people reflected on their experiences, and how collective insights emerged [66]. Rather than forming a group in the moment, the DFGs reactivated existing interpersonal ties, orientations and emotional atmospheres, extending their function beyond evaluation toward forms of unintended interventional engagement [36, 67]. These dynamics highlight a broader methodological point: DFGs produce data not only through what participants say, but through the relational, technological and emotional arrangements that constitute the setting. The DFGs thus become both a site of data collection and a temporary social intervention, especially in contexts where participants share therapeutic or programmatic histories [5, 68]. This is particularly relevant in medical and digital-health research, where qualitative components are often embedded in complex intervention contexts and where infrastructural and relational conditions shape how participants articulate their experiences [30]. The sociodemographic characteristics of the sample are not incidental to this methodological analysis: variables such as rurality and educational background are widely recognized indicators of differential digital access and digital literacy [69], while chronic illness and subjective health burden may influence participants’ capacity to engage with digitally mediated interaction and navigate technical challenges during the sessions. These characteristics thus directly inform the interactional and infrastructural conditions analyzed in this study and should be understood as methodologically relevant dimensions of the research situation rather than descriptors carried over from the parent evaluation study. By reflecting on DFGs as relational, contextual and socio-technical encounters, this study contributes to ongoing methodological debates about how qualitative knowledge is produced in digitally mediated health research [6, 7, 44]. Several of the dynamics observed in our study including pre-existing cohort cohesion, peer-supported sense-making, and unintended interventional effects are well-documented features of group-based qualitative research more generally and are not exclusive to the digital format [70–72]. This distinction is analytically important: we do not claim that these phenomena are caused by digitalization. Rather, our argument is that the digital setting distinctively reconfigures, mediates, and in some cases intensifies these dynamics through platform affordances, technologically structured turn-taking, device-dependent visibility, and the particular interactional demands of the online environment. The contribution of this study lies precisely in tracing how a familiar set of relational and methodological dynamics takes on a specific shape when transposed into a digital, intervention-linked research context. At the same time, not all dimensions identified in this study relate equally to the digital format itself. While dynamics such as cohort cohesion, peer-supported reflection, and unintended supportive effects are likely to emerge in many longitudinal or intervention-linked focus group settings, regardless of format, other observations were more directly tied to the conditions of digitally mediated interaction. In particular, platform-dependent visibility, technologically structured turn-taking, infrastructural instability, device-related asymmetries, and the expanded role of researchers as facilitators of both interaction and technical coordination emerged as more directly linked to the digital conditions of the research situation. These dimensions illustrate more specifically how digital environments reorganize participation, interactional flow, and the practical conditions under which qualitative data are generated. Distinguishing between generic FG effects and format-specific socio-technical influences is therefore essential for a nuanced interpretation of digitally mediated qualitative data. However, the digital setting reconfigured these established interactional processes by mediating visibility, turn-taking, and participation through platform affordances and infrastructural conditions. Thus, rather than creating entirely new forms of interaction, the online environment shaped and intensified familiar FGs dynamics in distinctive ways, for instance through technologically structured turn allocation, altered nonverbal communication, and the emergence of digital differences. The expanded role of researchers in the online environment of digital health research illustrates this point [6, 8, 68, 73]. Such configurations underline that researchers in digital qualitative work function as part of the setting’s infrastructure [7, 16, 74, 75].

At the same time, the DFGs occasionally generated supportive, reflective or motivational dynamics that resembled elements of the group approach within the psychosocial intervention itself. These unintended interventional moments align with broader observations in qualitative health research that interview and FGs encounters may elicit unintended emotional or therapeutic effects, particularly when participants share illness trajectories or therapeutic histories [76–79]. Guillemin and Gillam [80] describe such instances as ethically important moments, in which research interactions produce significance beyond the intended evaluative task. In our study, the DFGs appeared to intensify these dynamics by reactivating relational ties formed during the initial inpatient phase of the intervention and by affording brief experiences of reassurance, validation or shared struggle. These observations highlight that the boundary between unintended benefit/unintended effects in qualitative research embedded in interventions can become blurred [19, 20, 22, 77, 81, 82]. This raises methodological and ethical considerations for studies embedded in clinical or psychosocial interventions: FGs may document change, but under certain conditions they may also participate in shaping it. Reflexive attention to these unintended dynamics is therefore essential when using qualitative methods to inform or evaluate health interventions.

The dynamics observed in our DFGs also resonate with debates in participatory and co-creative research traditions, where group discussions are understood not merely as data-collection tools but as social spaces that enable shared meaning-making, mutual learning and forms of collective agency [83–85]. Participatory studies have shown that FGs can produce dialogical, empowerment-oriented interactions in which participants shape agendas, validate each other’s experiences and generate insights collaboratively rather than individually [86, 87]. While our study did not follow a participatory design, the relational cohesion and peer-supported reflection visible in the sessions echo such traditions and highlight how FGs may temporarily assume co-creative qualities [5, 42, 88].

Together, these insights highlight the methodological value of treating DFGs as relational and contextual events, especially in the context of health services, public health, medical, and interventional/evaluation research. Recognizing how socio-technical arrangements shape participation and meaning-making strengthens the interpretive robustness of qualitative components in medical research. Overall, our findings contribute to methodological innovation by integrating socio-technical and socio-material perspectives with interactional approaches to examine how DFGs shape the production of qualitative data. The socio-technical lens illuminates how platform affordances and digital infrastructures structure participation; the socio-material perspective draws attention to the constitutive role of devices and connectivity as non-human actors in the research situation. We argue that DFGs constitute socio-technical constellations in which human actors, technological infrastructures and interventional histories jointly shape meaning-making processes. This conceptualization expands existing methodological frameworks for qualitative data collection in digital, clinical and mixed-methods research. In that manner, our findings suggest that the choice between digital and in-person FGs should not be guided by pragmatic considerations alone, but by the relational, technological and institutional conditions of the research situation. This aligns with comparative methodological research showing that while both formats can generate rich qualitative data, they differ in how interaction, participation and meaning-making are shaped by the setting [22, 70, 71]. DFGs are particularly suitable when participants are already embedded in digitally mediated environments, when accessibility and continuity of participation are central, and when socio-technical arrangements are themselves part of the phenomenon under study. In contrast, in-person FGs may be preferable in contexts where embodied co-presence, nuanced non-verbal communication and the establishment of trust in newly formed groups are crucial. Rather than treating the two formats as interchangeable, researchers should therefore consider how different configurations of participants, topics and infrastructures shape interaction, participation and data quality.

### Implications for qualitative research and digital inquiry

The findings of this study highlight several methodological implications for the use of DFGs in medical and health research.

*First*, the presence of pre-existing cohort relationships demonstrates that DFGs cannot be understood solely as neutral data-collection tools, as they are embedded in the relational and procedural histories of interventions. This has concrete implications for how sampling, group composition, and timing are conceptualized in qualitative components of study designs. Researchers should consider whether participants share prior relational histories, at what point in the intervention trajectory the DFG is scheduled, and how these conditions will shape the interactional dynamics and the range of perspectives that can be articulated in the session. Where pre-existing cohort relationships are present, they should be treated as an analytical condition to be documented and interpreted rather than a confound to be controlled.

*Second*, the unintended interventional dynamics observed in our sessions including peer-supported learning, motivational reinforcement, reflective self-regulation, and affective containment indicate that DFGs may inadvertently reactivate behavioral processes tied to the intervention. For evaluations of digital or hybrid health programs, researchers should therefore consider whether DFGs might not only document change but also influence it. This has implications for the timing of qualitative data collection within intervention studies, for informed consent procedures that acknowledge potential therapeutic effects of participation, and for the interpretation of qualitative data collected in close temporal proximity to the intervention itself.

*Third*, the analysis shows that platform characteristics, device differences, and interactional rhythms directly shape the texture and depth of digital qualitative data. This underscores the need for explicit methodological decisions regarding platform choice, technical preparation, and facilitation design. DFGs require stable infrastructural conditions and careful facilitation to maintain participation quality and interactional balance. Researchers should document the technical conditions of each session as part of the methodological record, and should consider how platform affordances (including turn-taking structures, visibility settings, and recording functions) constitute rather than merely mediate the research situation.

*Fourth*, the digital divide remains an important structural constraint. Variations in digital literacy, connectivity, and equipment can affect who participates, how continuously individuals remain present, and how confident they feel in the conversation. These factors can introduce systematic biases in sample composition and data quality in studies relying on digital qualitative methods. Preparatory measures such as technical instruction sheets and individual test runs, while valuable, cannot fully mitigate these effects. Researchers should actively document device and connectivity differences, consider their implications for the generalizability of the data, and design facilitation practices that accommodate differential levels of digital access.

*Fifth*, the expanded role of researchers in digital settings, combining facilitation, technical guidance, and emotional attunement, demands reflexive awareness and explicit attention in methodological reporting. Online qualitative research in medical or health services contexts requires researchers to actively mediate between technological affordances, interactional cues, and participants’ emotional needs. This raises practical questions about role distribution between co-facilitators, methodological training for digital qualitative work, and the visibility of researcher influence in data generation. Reflexive documentation of researcher interventions (including technical troubleshooting, turn allocation or emotional anchoring) should be considered an integral part of the audit trail in digital qualitative research.

### Limitations

This study has several limitations that should be considered when interpreting its methodological contribution. First, the DFGs were conducted with established intervention cohorts, which represent an intended analytical condition rather than a methodological limitation. The study therefore speaks most directly to digital qualitative encounters situated within ongoing or recently completed group-based interventions. As participation in DFGs depends on access to suitable devices, stable internet connections and basic digital literacy, the interactional field is shaped by those who are able to enter and remain in the digital environment. While this constitutes part of the socio-technical conditions under examination, the study did not systematically assess reasons for non-participation. The study design did not include a comparison with in-person FGs or alternative qualitative formats. As a result, the specificity of certain dynamics to the digital format cannot be definitively established. In particular, the dynamics of pre-existing cohort cohesion and unintended interventional effects are likely to arise in any longitudinal or cohort-based FG design, digital or otherwise. The present study documents how these dynamics manifest and are shaped within a digital setting and not that they are caused by it. A further limitation concerns the empirical basis of the methodological claims advanced in this paper: while the dynamics described were observable across multiple DFG sessions and participants, the dataset nonetheless remains limited in scope, and the patterns identified should be understood as analytically meaningful rather than statistically representative; replication across a broader range of digital focus group settings would be needed to substantiate these claims more fully. Future comparative studies contrasting digital and in-person FGs within the same intervention context would be valuable for disentangling format-specific from design-specific effects. Overall, the conclusions relate to digitally mediated qualitative research embedded in structured intervention contexts and should be transferred with caution to settings that differ substantially from these conditions.

## Conclusion

This study shows that DFGs embedded in a hybrid psychosocial intervention are shaped by socio-technical, relational and procedural conditions that actively influence qualitative data production. Cohort cohesion, unintended interventional dynamics, platform affordances, digital access conditions and expanded researcher roles affected the production of qualitative data. Recognizing these dynamics is essential for designing and interpreting digital qualitative methods in medical research. Grounded in empirical examples, the study provides guidance for conducting rigorous and equitable DFGs in digitally mediated health contexts.

## Data Availability

The datasets used are not publicly available due to the privacy regulations of the project but are available from the corresponding author upon reasonable request.

## Abbreviations

COREQ: Consolidated criteria for reporting qualitative research
DFGs: Digital Focus Groups
FG-ID: Digital Focus Group identification number
FGs: Focus Groups
I: Interviewer (of the DFGs)
ISCED: International Standard Classification of Education
Max.: Maximum
Min: Minimum
P: Participant (of the DFGs)
PC: Personal computer
PE³PP: German acronym of the Project
Pos: Position within the transcript
SiA: Situational Analysis
TUM: Technical University of Munich
